# Cp_2_TiCl/D_2_O/Mn, a formidable reagent for the deuteration of organic compounds

**DOI:** 10.3762/bjoc.12.154

**Published:** 2016-07-25

**Authors:** Antonio Rosales, Ignacio Rodríguez-García

**Affiliations:** 1 Department of Chemical and Environmental Engineering, Escuela Politécnica Superior, University of Sevilla, 41011 Sevilla, Spain; 2 Petrochemical Engineering, Universidad de las Fuerzas Armadas-ESPE, 050150 Latacunga, Ecuador; 3 Química Orgánica, CeiA3, Universidad de Almería, 04120 Almería, Spain

**Keywords:** deuteration, deuterium atom transfer, radical and/or organometallic chemistry, titanocene

## Abstract

Cp_2_TiCl/D_2_O/Mn is an efficient combination, sustainable and cheap reagent that mediates the D-atom transfer from D_2_O to different functional groups and can contribute to the synthesis of new deuterated organic compounds under friendly experimental conditions and with great economic advantages.

## Introduction

Deuterium is a stable isotope of hydrogen with 0.015% natural abundance broadly used in organic chemistry, pharmacology, organometallic chemistry, spectroscopy and many other fields [1–4]. Exchange of hydrogen for deuterium produces primary and secondary kinetic isotope effects (KIE) causing isotopically substituted molecules to react at different rates (*k*_H_ ≠ *k*_D_). This behaviour is due to the differences in bond dissociation energies for both species, which in turn, is dependent upon the zero point for the vibrational energy of both isotopic molecules. As the mass of deuterium is about twice the mass of hydrogen there is a larger activation energy for the C–D bond dissociation than for the C–H bond [4]. The KIE observed allows multiple applications of the deuterated compound such as the enhancement of the metabolic stability of pharmaceutical drugs, the use of internal standards for mass spectrometry, the elucidation of biosynthetic pathways, and the study of reaction mechanisms and selectivity control reactions.

In an effort to develop efficient procedures for the preparation of deuterated compounds, several methodologies of deuteration have been reported [5]. One of the first procedures reported was the acid- or base-catalyzed exchange of enolizable protons for deuterium. However, in order to achieve high isotopic purities through this procedure, multiple treatments of the enolizable substrate with deuterium oxide are required. Also, this method is not suitable for the incorporation of deuterium at not enolizable positions [6]. Later, the reduction of functional groups using deuterated reagents emerged as a powerful tool for deuteration [7]. The principal disadvantage of the use of reducing agents labelled with deuterium is the high cost of these reagents and the handling of highly flammable substances. The use of palladium metal and D_2_O is a useful and efficient methodology for H/D exchange in aliphatic and benzylic C–H bonds [8–9]. More recently, organometallic catalysts have been used in the development of methods for deuteration of organic compounds. In this sense, it has been reported that iridium complexes can catalyse the H/D exchange of arenes, cyclic alkenes and vinyl groups [10–12]. Ruthenium complexes catalyse α-deuteration of amines and alcohols [13] and palladium complexes catalyse the *ortho*-selective deuteration of arenes [14]. Also, SmI_2_/D_2_O-mediated the chemoselective synthesis of α*,*α-dideuterio alcohols directly from carboxylic acid under single-electron-transfer conditions [15]. However, many of these procedures are too specific, being useful only for a particular functional group while the synthesis of the catalysts are very laborious and costly.

## Discussion

In this paper we summarize the applications of Cp_2_TiCl/Mn for the deuteration of organic compounds using D_2_O as deuterium atom donor.

**Scheme 1:**
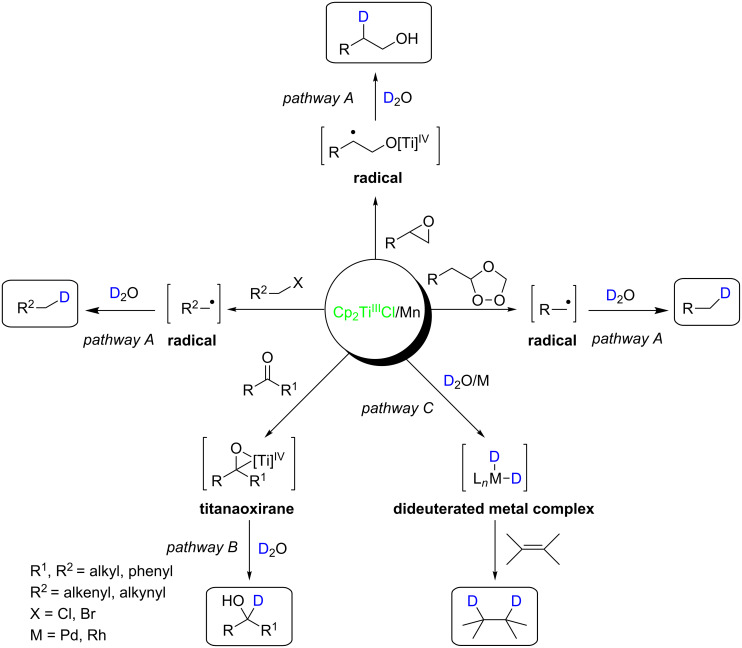
Formation of reaction intermediates susceptible of being reduced by Cp_2_TiCl/Mn/D_2_O.

Cp_2_TiCl, consists of titanium, one of the most abundant transition metals in the Earth’s crust [16], that can be easily prepared from commercial Cp_2_TiCl_2_ by using reductants such as Mn, Zn or Al [17–18], generating in THF, in absence of water a green solution, or a blue one in the presence of water. This complex is a single electron transfer system (SET) that has an unpaired d*-*electron and a vacant site, allowing heteroatoms with free valence electrons to coordinate and undergo electron transfer through an inner-sphere mechanism to generate carbon radicals or intermediate titanaoxiranes (Scheme 1). This SET is capable of promoting and/or catalyzing several transformations in organic chemistry [17–25]. One of the most relevant transformations is the H/D-atom transfer from H_2_O/D_2_O to carbon radicals (pathway A) (obtained from epoxides [26–28], ozonides [29] or activated halides [30] and Cp_2_TiCl/Mn), to intermediate titanaoxiranes (pathway B) [31–32] (obtained from carbonyl compounds and Cp_2_TiCl/Mn), and to late transition metals (pathway C) [33] in a process mediated by Cp_2_TiCl/Mn/H_2_O or D_2_O which allows for the reduction of alkenes or alkynes (Scheme 1).

**Scheme 2:**
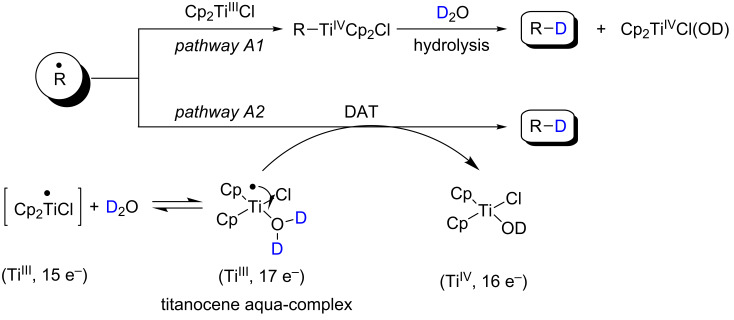
Proposed reduction of radicals via hydrolysis of an organometalic alkyl-Ti^IV^ or as DAT.

In presence of D_2_O these radicals (pathway A) can be reduced into deuterated compounds. The reduction can proceed via hydrolysis of an organometallic alkyl-Ti^IV^ intermediate (Scheme 2, pathway A1) or via deuterium-atom transfer (DAT) from D_2_O to radicals (Scheme 2, pathway A2). In the case of the intermediate titanaoxirane (pathway B) D_2_O could promote the hydrolysis to generate the deuterated compound.

DAT from D_2_O to radicals can be explained on the basis of the paper reported by Oltra and Rosales et al. [26–27]. In this paper, to explain HATs from water it was proposed that the co-ordination of water to Cp_2_TiCl might weakens the strength of the O–H bond. In this way a single electron transfer from titanium to oxygen might facilitate the HAT from the titanocene aqua-complex to the free radicals. Theoretical calculations supported that the coordination of water to Cp_2_Ti^III^Cl weakens the O–H bond, indicating a bond-dissociation energy (BDE) for the intermediate aqua-complex of only 49 kcal/mol. This points to a decrease of almost 60 kcal/mol compared to the calculated BDE of water. Later, Gansäuer et al. proposed a modified structure of the intermediate aqua-complex on the basis of cyclic voltammetry, theoretical calculations and electro-paramagnetic resonance techniques studies [28,34]. These results are in agreement with the previously reported results by Wood et al. [35] and Renaud et al. [36] describing the effect of complexation with a Lewis acid on the strength of the O–H bond in water.

Although more theoretical and experimental studies should be performed to determine the mechanism of reduction of radicals using Cp_2_TiCl and water, it can be stated that tertiary and hindered radicals are normally reduced via HAT from water in a process mediated by Cp_2_Ti^III^Cl. Primary and unhindered radicals are normally reduced via hydrolysis of an organometallic alkyl-Ti^IV^ intermediate [37].

This HAT or protonation mechanism by Cp_2_TiCl/D_2_O/Mn, compared with the single-electron-transfer conditions using SmI_2_/D_2_O in the synthesis of α*,*α-dideuterated alcohols from carboxylic acids, does not require the activation of the organometallic species with base and substoichiometric amounts of Cp_2_TiCl can be used.

Deuteration of alkenes/alkynes [14] using Cp_2_TiCl/D_2_O/Mn and late transition metals (pathway C) was rationalized suggesting that the aqua-complex intermediate could facilitate the DAT from D_2_O to the late transition metal to give a metal dideuterated species, which accomplishes the deuteration of alkenes/alkynes.

**Scheme 3:**
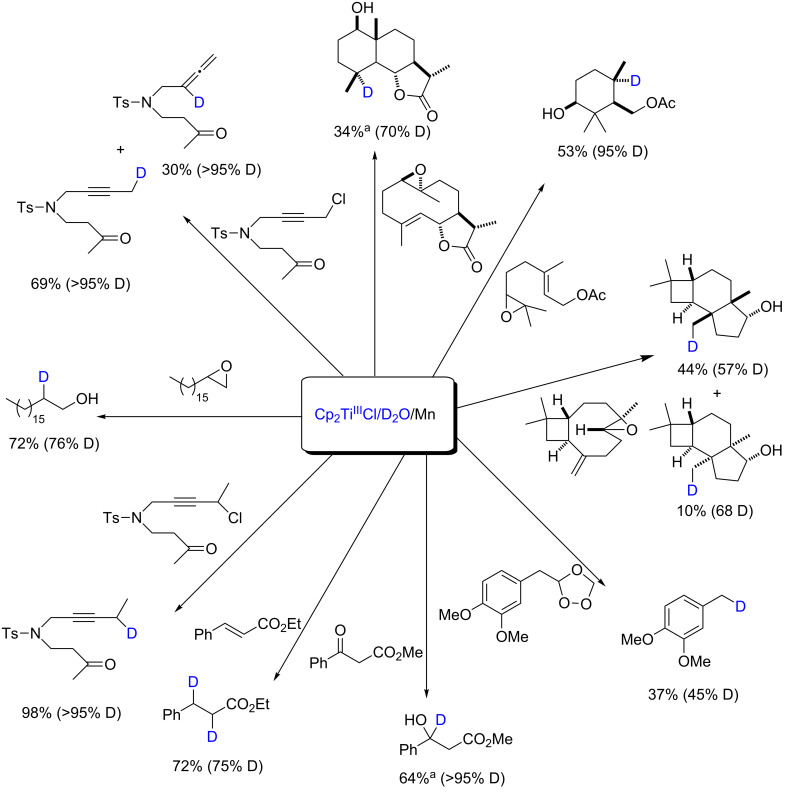
Examples of deuterations of organic compounds using Cp_2_TiCl/D_2_O/Mn. ^a^Substoichiometric amount of Cp_2_TiCl; D: deuterium incorporation.

In any case, apart from mechanistic considerations, the Cp_2_TiCl/D_2_O/Mn mixture has emerged as an excellent reagent for the deuteration of organic compounds from epoxides [2,27,37], ozonides [29], ketones [31–32], activated halides [30–32], alkenes and alkynes [33]. Several examples are presented in Scheme 3.

The results show that the combination Cp_2_TiCl/D_2_O/Mn is able to promote and/or catalyze deuteration of organic compounds by reduction or radical cyclization using reagents that are cheap, abundant and environmentally friendly. Certainly, this new methodology of deuteration will contribute to the synthesis of new deuterated organic compounds with applications as internal standards, pharmaceutical drugs and new materials, among others.

## Conclusion

In summary, we presented an overview of the Cp_2_TiCl/D_2_O/Mn combination as an efficient, cheap, selective, and sustainable reagent compatible with different functional groups that mediates the deuteration of organic compounds from epoxides, ozonides, carbonyl compounds, activated halides, alkenes and alkynes, under mild and environmentally safe reaction conditions. We foresee that in the near future other complexes of Ti^III^ will be used for the deuteration of organic compounds.
